# SYT4 Interacts with PSMC6 to Facilitate Malignant Progression in Gastric Carcinoma via Activating Wnt/β-catenin Signaling

**DOI:** 10.7150/ijbs.118672

**Published:** 2025-10-20

**Authors:** Wen Huang, Rongkui Luo, Huimei Wang, Shuo Yang, Zixiang Yu, Yufeng Liu, Huaiyu Liang, Yanyan Shen, Xiaolei Zhang, Licheng Shen, Sujie Akesu, Chen Xu, Yingyong Hou

**Affiliations:** 1 Department of Pathology, Zhongshan Hospital, Fudan University, Shanghai 200032, China.; 2 Department of Orthopaedics, Tongzhou Bay People's Hospital, Nantong, Jiangsu 226000, China.; 3 Department of Orthopaedics, Nantong First People's Hospital, Affiliated Hospital 2 of Nantong University, Nantong, Jiangsu 226000, China.; 4 Shanghai Institute of Materia Medica, Chinese Academy of Sciences, Shanghai 201203, China.

**Keywords:** synaptotagmin-4 (SYT4), gastric cancer, PSMC6, Wnt/β-catenin signaling pathway, borussertib, drug target

## Abstract

**Background:** Gastric cancer (GC), a prevalent and life-threatening malignancy, poses significant challenges in diagnosis and prognosis due to its complex molecular pathogenesis. Identifying novel biomarkers and therapeutic targets is crucial for advancing treatment strategies and improving patient outcomes. This study investigates the role of synaptotagmin-4 (SYT4), recently identified as an oncogene, in GC development.

**Methods:** We integrated proteomic and clinical analyses to evaluate SYT4 expression levels and their correlations with clinical features. Bioinformatic and clinicopathological assessments further validated SYT4's clinical relevance. Through comprehensive *in vitro* and *in vivo* experiments—including immunoprecipitation-mass spectrometry (IP-MS), co-immunoprecipitation (Co-IP), GST pull-down assays, and TOP/FOP luciferase reporter assays—we delineated SYT4's biological functions and interaction mechanisms. Additionally, we investigated the therapeutic potential of borussertib, a specific SYT4 inhibitor, in suppressing GC tumorigenicity.

**Results:** SYT4 expression was significantly upregulated in GC tissues and strongly correlated with poor prognosis. Functionally, SYT4 drove cell proliferation, promoted cell cycle progression, and suppressed apoptosis in both cellular and animal models. Mechanistic investigations revealed that SYT4 directly interacts with PSMC6 via its C2B domain (amino acids 288-423), and stabilizes PSMC6 protein, thereby activating the Wnt/β-catenin signaling pathway. Notably, borussertib, a targeted SYT4 inhibitor, markedly suppressed SYT4 activity, leading to attenuated GC progression.

**Conclusion:** Our findings demonstrate that SYT4 is a critical driver of GC progression via activation of the Wnt/β-catenin pathway. Moreover, we uncovered a novel mechanism by which borussertib selectively inhibits SYT4's oncogenic activity, providing compelling evidence for its therapeutic potential in gastric cancer treatment.

## Introduction

Gastric cancer (GC), a leading malignancy in the digestive system, accounts for nearly 9% of all cancer-related deaths globally 1. Despite advancements in therapeutic approaches, the prognosis for GC patients remains grim, primarily due to the incomplete understanding of its molecular pathogenesis 2. While molecular biology has facilitated the discovery of several potential biomarkers and driver genes, their translation into clinical applications for GC has been limited 3. Consequently, the identification of effective biomarkers for GC is essential to enhance early diagnosis, prognosis assessment, and the development of novel therapeutic strategies.

Synaptotagmin-4 (SYT4), a member of the membrane protein family, primarily functions as a calcium sensor and regulator of exocytosis 4. Characterized by two C2 domains with internal repeats, SYT4 exhibits distinct Ca^2+^-dependent and -independent activities 5. Previous studies have implicated SYT4 in various physiological processes, including pancreatic β-cell maturation, distribution of dense-core vesicles in hippocampal neurons, and neuroprotection 6-8. Moreover, SYT4 has been reported to play an oncogenic role in cancer progression through genetic alterations. For instance, Qiong et al. 9 demonstrated that SYT4 overexpression promotes dendritic extension and melanoma cell activity via crosstalk mechanisms. A recent study also highlighted an association between SYT4 expression and the prognosis of GC patients 10. However, the exact role of SYT4 in GC, its molecular mechanisms, and its potential as a prognostic or therapeutic target remain to be fully elucidated.

In this study, we investigated SYT4 expression in GC tissues and its correlation with clinicopathological features and patient prognosis. Our *in vitro* and *in vivo* experiments revealed that SYT4 accelerates GC cell proliferation, promoted cell cycle progression, and suppresses apoptosis. Mechanistically, SYT4 interacts with proteasome 26S subunit ATPase 6 (PSMC6) through its C2B domain, leading to the activation of the Wnt/β-catenin signaling pathway. Furthermore, we identified borussertib, a covalent allosteric inhibitor of SYT4, which selectively suppresses SYT4-driven tumor growth in GC cells and xenograft models by inhibiting the Wnt/β-catenin pathway 11. Our results indicate that SYT4 is a promising biomarker and a potential therapeutic target for GC.

## Materials and Methods

### Sample preparation

The formalin-fixed, paraffin-embedded (FFPE) specimens from 90 GC patients were collected from Zhongshan Hospital, Fudan University, for proteomic characterization of gastric cancer. The methods for sample pretreatment, proteomic measurement, and analysis were conducted in accordance with our previous studies 12, 13. Additionally, clinicopathological analyses were performed on 1105 paired normal gastric tissues and 1429 primary GC tissues obtained between January 2014 and December 2020. All participants provided written informed consent, and the study protocol was approved by the Institutional Review Board (IRB) of Zhongshan Hospital, Fudan University.

### Differential expression analysis in GC

To identify differentially expressed proteins between gastric adenocarcinoma and para-cancerous tissues, we conducted Student's t-tests. Proteins were classified as upregulated or downregulated in GC using a threshold of FDR < 0.05 and |Log2FC| > 2. The Random Forest (RF) algorithm was employed to select significant features for classification based on feature importance. Lasso-Cox regression was applied to identify features with substantial impacts on the target variable by setting the coefficients of less important features to zero, thereby enhancing prediction accuracy. The prognostic relevance of these key proteins was further validated through Cox regression analysis.

### Immunohistochemistry (IHC)

The expression levels of SYT4 in paired normal and GC tissues were evaluated using the EnVision two-step staining method. Tissue microarrays were incubated overnight at 4°C with a rabbit monoclonal anti-SYT4 antibody (dilution 1:600; Invitrogen, Carlsbad, CA). Subsequently, the samples were incubated with a secondary antibody at room temperature for 30 minutes. Diaminobenzidine (DAB) was used as the chromogen for result visualization. Staining intensity and extent were independently assessed by two pathologists in a blinded manner. Staining intensity was graded on a scale of 0 to 3: 0 (no staining), 1 (weak), 2 (moderate), and 3 (strong). The final score was determined by multiplying the intensity score by the percentage of positively stained cells.

### Cell culture and transfection

The human GC cell lines (HGC27, MGC803, AGS, MKN45, and BGC823) and GES-1 cells (an immortalized normal gastric epithelial cell line) were obtained from the Cell Bank of the Shanghai Institute of Cells. All cell lines were cultured in DMEM (Biosharp, Hefei, Anhui, China) supplemented with 10% fetal bovine serum (Gibco, NE, USA), 100 U/mL penicillin, and 100 μg/mL streptomycin (Gibco, NE, USA) at 37 °C in a 5% CO2 atmosphere. Lentiviruses containing transgenes were purchased from GeneChem (Shanghai, China), with lentiviruses carrying an empty vector used as negative controls (NC). The constitutively active β-catenin mutant (S33Y) 14 was obtained from Cell Researcher Biotech (Shanghai, China). Following transfection, stable cell lines were established through puromycin or geneticin selection. Target sequences for shRNAs are provided in Supplementary Table S1.

### Quantitative real-time transcription PCR (qRT‒PCR) and western blotting (WB)

Total RNA was extracted from cell lines using TRIzol reagent (Beyotime Biotechnology, Haimen, China). RNA samples were reverse-transcribed into complementary DNA (cDNA) using a reverse transcription kit (Takara, Dalian, China). Quantitative PCR was performed on a QuantStudio 5 instrument (Applied Biosystems) to quantify cDNA levels. Relative gene expression was calculated using the 2-ΔΔCT method, with β-actin as the internal control. The qPCR primer sequences are listed in Supplementary Table S2.

For protein extraction, cultured cells were lysed using RIPA buffer (Beyotime) supplemented with phenylmethylsulfonyl fluoride (PMSF) for 20 minutes. Protein concentrations were determined using a BCA protein assay kit (Beyotime). Nuclear and cytoplasmic proteins were separated using the NE-PER Nuclear and Cytoplasmic Extraction Reagents kit (Thermo Scientific). Proteins were resolved by SDS‒PAGE on a 4‒20% precast gel and transferred onto a PVDF membrane. The membrane was incubated overnight at 4°C with primary antibodies, followed by a 1‒hour incubation with secondary antibodies. Details of the antibodies used are provided in Supplementary Table S3.

### Protein stability test

To detect the stability of PSMC6 protein, SYT4-KD and control vector- transfected cells were treated with 50 µg/ml protein synthesis inhibitor cycloheximide (CHX), purchased from MedChemExpress company, for durations as 0, 2, 4 and 8 hours. Protein was extracted for Western blot.

### Cell counting assay Kit-8 (CCK-8)

Cells were plated in 96-well plates at a density of 5×10^3^ cells per well with 100 µL of medium and incubated overnight. Subsequently, 10 µL of CCK-8 reagent (Dojindo, Kumamoto, Japan) was added to each well, followed by a 2-hour incubation at 37°C. Absorbance was measured at 450 nm using a Multiskan™ FC Microplate Photometer (Thermo Scientific).

### Cell apoptosis and cycle assay

Cell apoptosis and cycle analyses were performed using BD FACSAria™ III flow cytometers (BD Biosciences, Franklin Lakes, NJ). Apoptosis was assessed with an apoptosis detection kit (BD Biosciences, NY, USA). Briefly, cell pellets containing 1×10⁵ cells were resuspended in 100 μL binding buffer, followed by the addition of 5 μL PE Annexin V and 5 μL 7-AAD. After a 20-minute incubation in the dark, 400 μL binding buffer was added, and apoptosis analysis was conducted within one hour. For cell cycle analysis, 1×10⁶ cells were serum-starved overnight and fixed in 75% ice-cold ethanol at 4°C for 12-14 hours. Fixed cells were permeabilized, incubated with 0.5 mL PI/RNase solution (BD Biosciences) for 15 minutes, and analyzed using FlowJo software, version 10.4 (Tree Star Inc.), with data collected from 10,000 events per sample.

### Animal experiments

Animal experiments were approved by the Institutional Animal Care and Use Committee of Zhongshan Hospital, Fudan University. Four-six weeks old male C-NKG mice (NOD.Cg-Prkdc^scid^112rg^em1cye^/Cya), with carried Prkdc^scid^ gene mutation and II2rg gene knock-out, were obtained from Cyagen (Guangzhou, China). Cells were counted and subcutaneously injected into the mice at a dose of 5×10⁶ cells per mouse. Mice were divided into two groups (n=5 per group): one group received cells with SYT4 knockdown (KD), and the other served as a negative control (KD-NC). Tumor size was measured every three days using the formula: volume (mm³) = 0.5 × width² × length. After four weeks, mice were euthanized, and tumors were weighed. All procedures followed the National Institutes of Health's Guide for the Care and Use of Laboratory Animals.

### Immunofluorescence

Cells cultured in 6-well chamber slides (Servicebio, Hubei, Wuhan, China) were rinsed with PBS and fixed with 4% paraformaldehyde for 10-15 min at room temperature. After permeabilization with 0.1% Triton X-100 for 10 min, the samples were washed three times with PBS, blocked with 5% BSA, and incubated with primary antibody overnight at 4 °C. The next day, the cells were incubated with secondary antibody in the dark for 1 h at room temperature, and the nuclei were counterstained with DAPI for 5 min. Images were observed by a laser scanning confocal microscope (FV1000, Olympus, Japan).

### Coimmunoprecipitation (co-IP) assay and immunoprecipitation-mass spectrometry (IP-MS)

For coimmunoprecipitation (co-IP) assays, cell lysates were prepared using RIPA lysis buffer and centrifuged at 16,000 × g for 20 minutes. One milligram of protein from the supernatant was incubated with 10 μg of anti-Flag (Abcam) or IgG overnight at 4 °C. Protein A/G magnetic beads (Thermo Fisher Scientific, Waltham, USA) were used to immunoprecipitate the antigen-antibody complex. The mixture was rotated gently at 4 °C for 2 hours. Bound proteins were eluted by heating in SDS loading buffer and separated by SDS-PAGE. Potential SYT4-interacting proteins were identified via immunoprecipitation-mass spectrometry (IP-MS) analysis conducted by Orizymes Biotechnologies Co., Ltd, in Shanghai, China.

### GST pull down

*E. coli*-derived GST-SYT4 and His-PSMC6 fusion proteins were constructed and purified by Yeasen (Shanghai, China). Two hundred micrograms of GST-SYT4 protein or GST-control were incubated with anti-GST magnetic beads (Orizymes) for 2 hours. The GST-SYT4 protein-coupled beads were then incubated with His-PSMC6 protein overnight at 4°C. After washing to remove unbound proteins, the beads were boiled in loading buffer, and the eluted proteins were detected by Western blotting (WB).

### Protein-protein interaction prediction

The structures of PSMC6 (PDB ID: 8CVT) and SYT4 (generated by AlphaFold) were retrieved from the Protein Data Bank and UniProt databases, respectively. Protein-protein docking was performed using Zdock v.3.0.2. In the ZDOCK module, the receptor and ligand proteins were loaded, and the relevant parameters were set in the toolbar. The ZDOCK program was executed, and the complex structure with the highest Zdock score was selected for further analysis and visualized in PyMOL.

### TOP/FOP luciferase reporter assay

To assess the activity of the Wnt/β-catenin signaling pathway, a TOP/FOP flash assay was performed. HEK293T stable cell lines expressing SYT4-OENC, SYT4-OE-siNC, and SYT4-OE-siPSMC6 were co-transfected with pRLTK and TOP/FOP flash reporter constructs (Promega, USA). After 24 hours, luciferase activity was measured using the Dual-Luciferase Reporter Assay System. The ratio of TOP to FOP activity was calculated to determine the activation level of the Wnt/β-catenin signaling pathway.

### Compound screening

A library of 517 FDA-approved small-molecule compounds was obtained from Selleck (Shanghai, China) and detailed in Supplementary Table S4. Cells were seeded in 96-well plates at a density of 5,000 cells per well in 100 µL of medium. After overnight incubation, adherent cells were treated with the compounds at 10 µM for 48 hours. Cell viability was determined using a microplate reader and 50% inhibitory concentration (IC50) should be calculated by statistical analysis.

### Surface plasmon resonance (SPR)

Surface plasmon resonance (SPR) binding assays were conducted at 25°C using CM5 sensor chips on a Biacore T200 instrument (GE Healthcare), following the manufacturer's guidelines. Recombinant SYT4 protein, supplied by Yeasen (Shanghai, China), was immobilized onto the CM5 chip (Cytova). Borussertib was first dissolved in DMSO to prepare a 20 mM stock solution, then diluted in PBS buffer and flowed over the SYT4-coupled chip. The binding affinity was evaluated by calculating the dissociation constant (KD) for borussertib relative to the SYT4 protein using the kinetic analysis mode of Biacore T200 software (version 1.0). Data visualization and analysis were performed using GraphPad Prism, version 8.0.

### Dosing treatment

To confirm the screening results, borussertib was applied to cells in the exponential growth phase at a final concentration of 15 µM, aligning with its IC50 value. After 24 hours of treatment, cells were collected via flow cytometry for apoptosis and cell cycle analysis. For the colony formation assay, cells were suspended in culture medium with or without 15 µM borussertib and plated in 6-well plates at 700 cells per well. The medium was refreshed every three days. After 14 days, colonies were stained with 0.1% crystal violet and counted for quantification.

### Effect of borussertib on the xenograft tumor model

To evaluate the therapeutic potential of borussertib *in vivo*, we established a subcutaneous tumor xenograft model. In this model, mice were administered intraperitoneal injections of borussertib at a dosage of 20 mg/kg for five consecutive days, followed by a two-day break 11. This administration schedule was continued for a total of 35 days, after which the animals were sacrificed.

### Statistical analysis

Bioinformatic analyses were conducted using R software. All experiments were repeated three times to ensure reproducibility. Clinicopathological characteristics and survival outcomes were evaluated using logistic regression and the Kaplan‒Meier method, respectively. For correlation analysis, the Spearman rank correlation test was employed. Group comparisons were performed using GraphPad Prism 9 software.

## Results

### Elevated expression of SYT4 is associated with adverse prognosis in GC Patients

**Figure 1:**
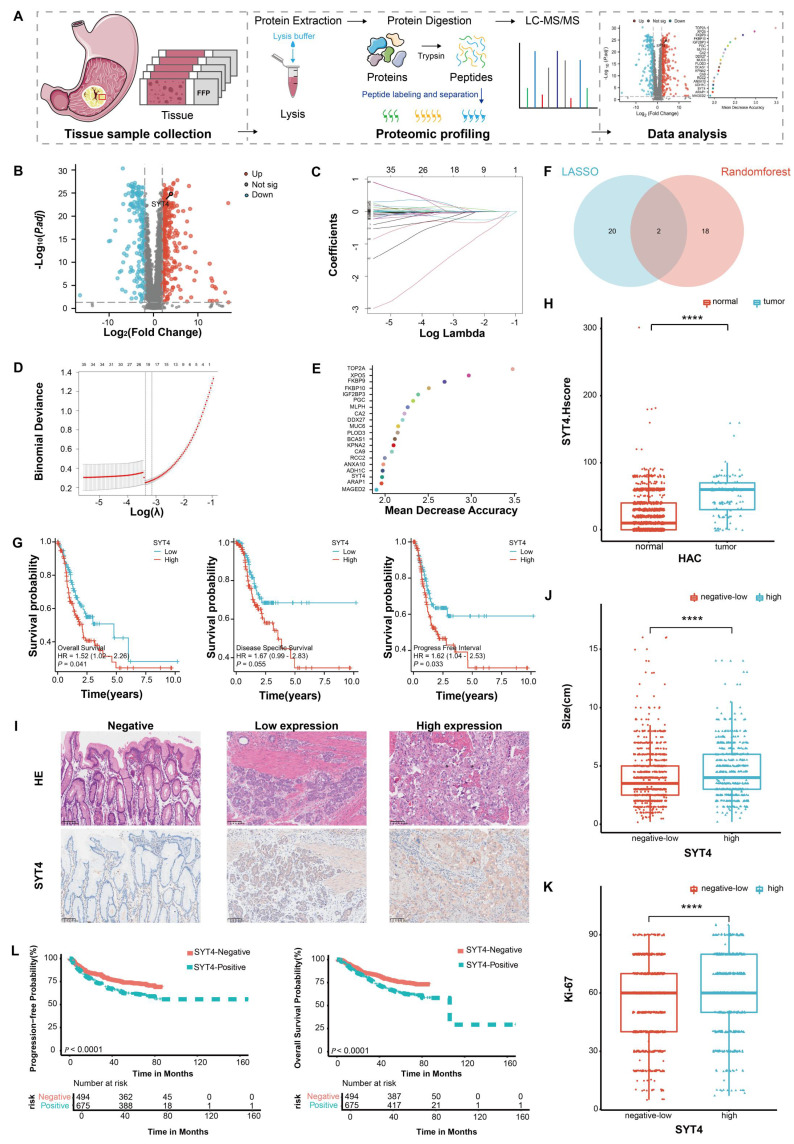
**Screening and verification of the potential biomarker in GC. A** The proteomics workflow involved 90 GC patients. **B** 478 differentially expressed proteins were identified using proteomic measurement. **C and D** Lasso-Cox regression analysis revealed 22 significant proteins. **E** The result of RF showed 20 significant proteins. **F** Venn plot displayed the intersection of the above two analysis. **G** SYT4 was negatively associated with survival in GC.** H and I** SYT4 expression was higher in GC tissues than in adjacent healthy tissues (Representative images of SYT4 staining, 200× magnification). **J** and** K** SYT4 expression was positively correlated with tumor size and Ki-67 proliferation index. **L** Correlation between PFS and OS with SYT4 expression in 1429 GC patients from our cohort.

In our pursuit to delineate the proteomic landscape and identify potential diagnostic biomarkers in GC, proteomic analysis based on intensity-based absolute quantification (iBAQ) was conducted on 90 GC samples alongside 77 paired gastric mucosa samples (Fig. 1A, with patients' baselines detailed in Supplementary Table S5). A comprehensive catalog of 3,885 proteins was detected across all samples. Notably, 478 proteins were flagged as differentially expressed (FDR<0.05, |Log2FC|>2), comprising 295 upregulated and 183 downregulated proteins in tumor tissues (Fig. 1B). To pinpoint proteins correlated with prognosis, we performed Lasso-Cox regression analysis and random forest (RF) analysis. As depicted in Figures 1C and 1D, 22 proteins (coef ≠ 0) were deemed significant through Lasso-Cox regression (Fig. 1E, highlighting the top 20 impactful proteins). Venn diagram analysis further intersected the results from these two approaches, unveiling SYT4 and FKBP9 as significant markers (Fig. 1F). Ultimately, survival analyses — assessing overall survival (OS), disease-specific survival (DSS), and progression-free interval (PFI) — firmly established SYT4 as a potential oncogene and prognostic predictor in GC (Fig. 1G and Supplementary Figure S1).

Immunohistochemistry (IHC) was applied to 1,429 primary GC tissue samples and 1,105 adjacent normal tissue samples. SYT4 levels were markedly elevated in GC tissues (61.09% of GC samples) compared to normal tissues (8.42% of normal samples), with a P-value < 0.0001 (Fig. 1H). Patients were stratified into high and low SYT4 expression groups (Fig. 1I, three representative images of SYT4 staining). Further analysis disclosed significant disparities between these groups across multiple clinical parameters. Specifically, variations in tumor size (Fig. 1J), Ki-67 index (a marker of cellular proliferation) (Fig. 1K), lymph node involvement, histological differentiation, tumor stage (T stage), perineural invasion, vascular invasion, and TNM stage (a holistic cancer staging system) were all substantial (*P* < 0.001, Table 1). Survival analysis further indicated that elevated SYT4 expression was markedly linked to shortened progression-free survival (PFS) and overall survival (OS) (*P*<0.0001, Fig. 1L). Collectively, these extensive analyses position SYT4 as a potential oncogene with significant prognostic value in GC.

### SYT4 regulates cell proliferation, cell cycle progression, and apoptosis* in vitro*

Given the promoting role of SYT4 in the progression of GC and its important function in treatment resistance 10, 15, 16, as well as its significant clinical association with GC, we speculated that SYT4 might function as an oncogene involved in GC progression. To test this hypothesis, we first compared the mRNA and protein levels of SYT4 in GC cell lines and the normal gastric epithelial cell line GES-1 using qRT-PCR and WB. The results showed that both the mRNA and protein levels of SYT4 were significantly higher in GC cell lines than in GES-1 cells, with the most notable differences observed in HGC27 (undifferentiated, with high metastasis potential, low adhesion and strong drug resistance) and MGC803 (poorly differentiated GC cell lines) cells (*P* < 0.01, Supplementary Figure S2A).

To further investigate the role of SYT4 in GC, we established SYT4-overexpressing (OE), SYT4-knockdown (KD), and corresponding control (NC) cell lines by infecting HGC27 and MGC803 cells with lentiviruses. The transfection efficiency was confirmed using qPCR and WB (Supplementary Figure S2B).

**Figure 2:**
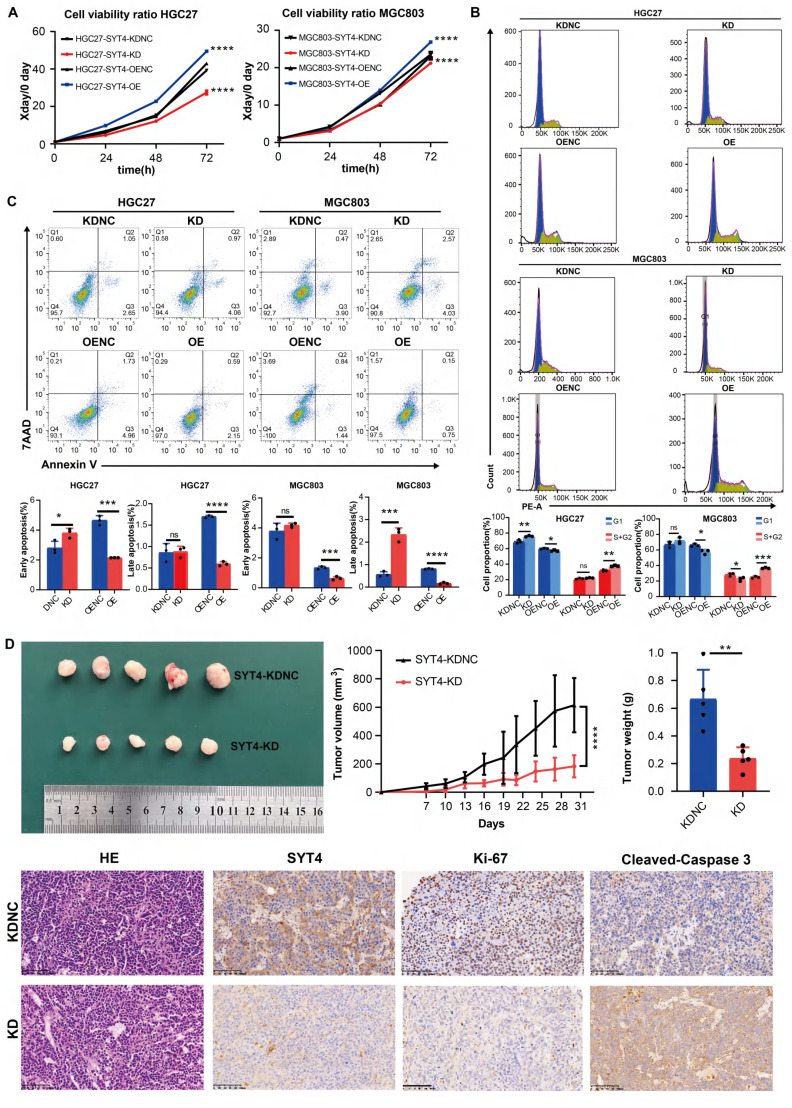
** Functional effects of SYT4 on GC cells. A** SYT4 knockdown suppressed cell proliferation, and SYT4 overexpression promoted proliferation. **B** Downregulation of SYT4 inhibited the G1 to S+G2 transition, while SYT4 overexpression blocked the G1 phase and enhanced the proportion of cells in the S+G2 phase. **C** SYT4 depletion induced GC apoptosis; SYT4 overexpression increased the cell apoptosis rate. **D** Tumor volume and tumor weight of control and SYT4 knockdown xenografts originating from MGC803 cells, as well as IHC staining of primary tumor tissues.

Subsequently, a series of assays were performed, including CCK-8, apoptosis, cell cycle, Transwell, and scratch assays. The transwell and scratch assays indicated that SYT4 overexpression did not affect the invasive and migratory abilities of HGC27 and MGC803 cells (Supplementary Figure S3). However, compared to the NC group, the SYT4-OE group exhibited significantly enhanced cell proliferation, while the SYT4-KD group showed reduced proliferation in both HGC27 and MGC803 cells (Fig. 2A).

Cell cycle analysis by flow cytometry revealed that in the HGC27-SYT4-KD group, the proportion of cells in the G1 phase was significantly increased compared to the NC group (*P* < 0.001). Conversely, in the SYT4-OE group, the proportion of cells in the G1 phase was decreased (*P* < 0.05), while the proportion of cells in the S+G2 phases was increased (*P* < 0.01) compared to the NC group (Fig. 2B). Similarly, in MGC803 cells, SYT4-KD led to a decrease in the percentage of cells in the S+G2 phases (*P* < 0.05), whereas SYT4-OE promoted the transition of cells from the G1 to S+G2 phases.

Regarding apoptosis, after SYT4 silencing, the HGC27-SYT4-KD group showed a significantly higher proportion of early apoptotic cells compared to the NC group (*P*<0.001). In contrast, both early and late apoptosis rates were markedly reduced in the HGC27-SYT4-OE group compared to the NC group (*P* < 0.05). Consistently, in MGC803 cells, the SYT4-KD group exhibited increased apoptosis rates, particularly in late apoptosis (*P* < 0.001), while the SYT4-OE group showed lower apoptosis rates in both early and late stages compared to the NC group (*P* < 0.001 and *P* < 0.0001, respectively) (Fig. 2C). In summary, these findings indicated that SYT4 promotes cell proliferation, lead to cell cycle progression, and reduces apoptosis in GC cells.

### SYT4 regulates GC growth* in vivo*

To elucidate the *in vivo* role of SYT4 in GC, we conducted experiments using a xenograft model. MGC803 cells with reduced SYT4 expression via knockdown (SYT4-KD) and control cells with normal SYT4 expression (SYT4-KDNC) were subcutaneously injected into C-NKG mice. The results demonstrated that the tumor size and weight in mice injected with SYT4-KD cells were markedly reduced compared to those in mice injected with control cells (Figure 2D). Immunohistochemical (IHC) analysis revealed that tumors from the SYT4-KD group showed lower SYT4 expression and reduced levels of Ki-67, a marker of cell proliferation, compared to the control group (Figure 2D). Collectively, these findings suggested that SYT4 silencing can effectively suppress the growth of GC cells *in vivo*, as evidenced by smaller tumor size and lower proliferative activity.

### SYT4 activates the Wnt/β-catenin signaling pathway

**Figure 3:**
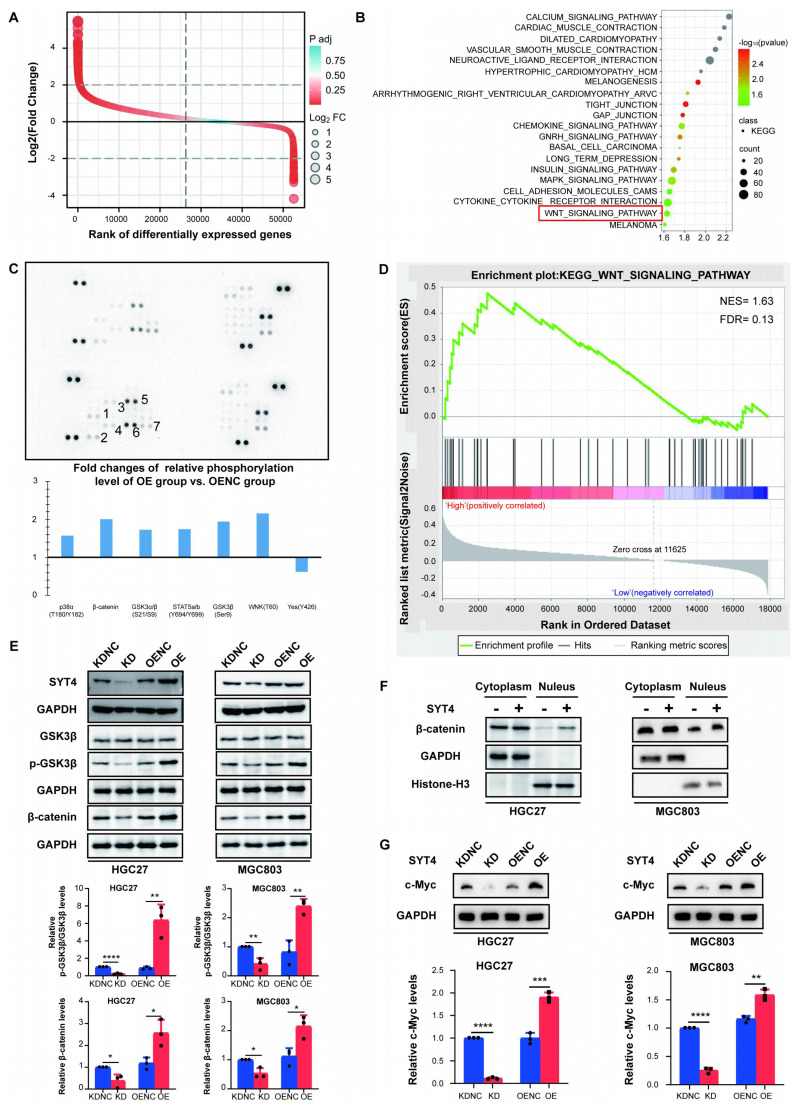
** SYT4 is involved in regulating the Wnt/β-catenin signaling pathway. A** and **B** GSEA enrichment analysis focused on KEGG pathways enriched in differentially expressed genes that related to SYT4 level. **C** The potential downstream pathway-related proteins detected by protein array analysis. **D** Wnt pathway was detected by GSEA analysis, with the normalized enrichment score (NES) of 1.63 and false discovery rate (FDR) of 0.13. **E** SYT4 expression increased the levels of p-GSK-3β/GSK-3β and β-catenin, and SYT4 knockdown showed the opposite effect on these markers. **F** In the SYT4-OE group, the β-catenin levels in the cell nucleus were notably elevated compared to those in the control group. GAPDH was used as the internal control for cytoplasmic proteins, whereas Histone-H3 for nuclear proteins. Alterations in the levels of the downstream target protein c-Myc were observed following SYT4 silencing/overexpression.

To further explore the underlying mechanisms of SYT4 in GC, we conducted Gene Set Enrichment Analysis (GSEA) and protein array studies to identify the downstream signaling pathways mediated by SYT4. Based on the differential analysis of SYT4 levels (Fig. 3A), GSEA was performed on the differentially expressed genes to identify pathways upregulated in association with SYT4 expression. Pathways with statistically significant differences (*P* < 0.05), including MAPK signaling pathway, cytokine receptor interaction and Wnt signaling pathway, are presented in Figure 3B. Protein array analysis further revealed that SYT4 expression correlates with the levels of GSK3β (Ser9), β-catenin, and WNK1 (Thr60) (Fig. 3C). Combining these results, we focused on the Wnt signaling pathway (Fig. 3D).

Further investigation into the interaction between SYT4 and the Wnt pathway revealed that SYT4 knockdown significantly decreased p-GSK3β levels, while SYT4 overexpression increased p-GSK3β levels, without affecting total GSK-3β protein levels (Fig. 3E). Consistently, SYT4 downregulation led to a significant reduction in β-catenin levels in MGC803 and HGC27 cells, whereas SYT4 upregulation had the opposite effect (Fig. 3E).

To determine whether SYT4 upregulation promotes the nuclear translocation of β-catenin, we analyzed nuclear protein extracts. Results of western blot showed that β-catenin levels in the nucleus were significantly higher in the SYT4-overexpressing group compared to the control group (Fig. 3F). Additionally, the expression of c-Myc, a target gene of β-catenin, increased with SYT4 upregulation and decreased with SYT4 downregulation (Fig. 3G). Collectively, these findings indicated that SYT4 plays a critical role in regulating the Wnt/β-catenin signaling pathway in GC, potentially driving tumor progression through this mechanism.

### SYT4 directly interacts with PSMC6

**Figure 4:**
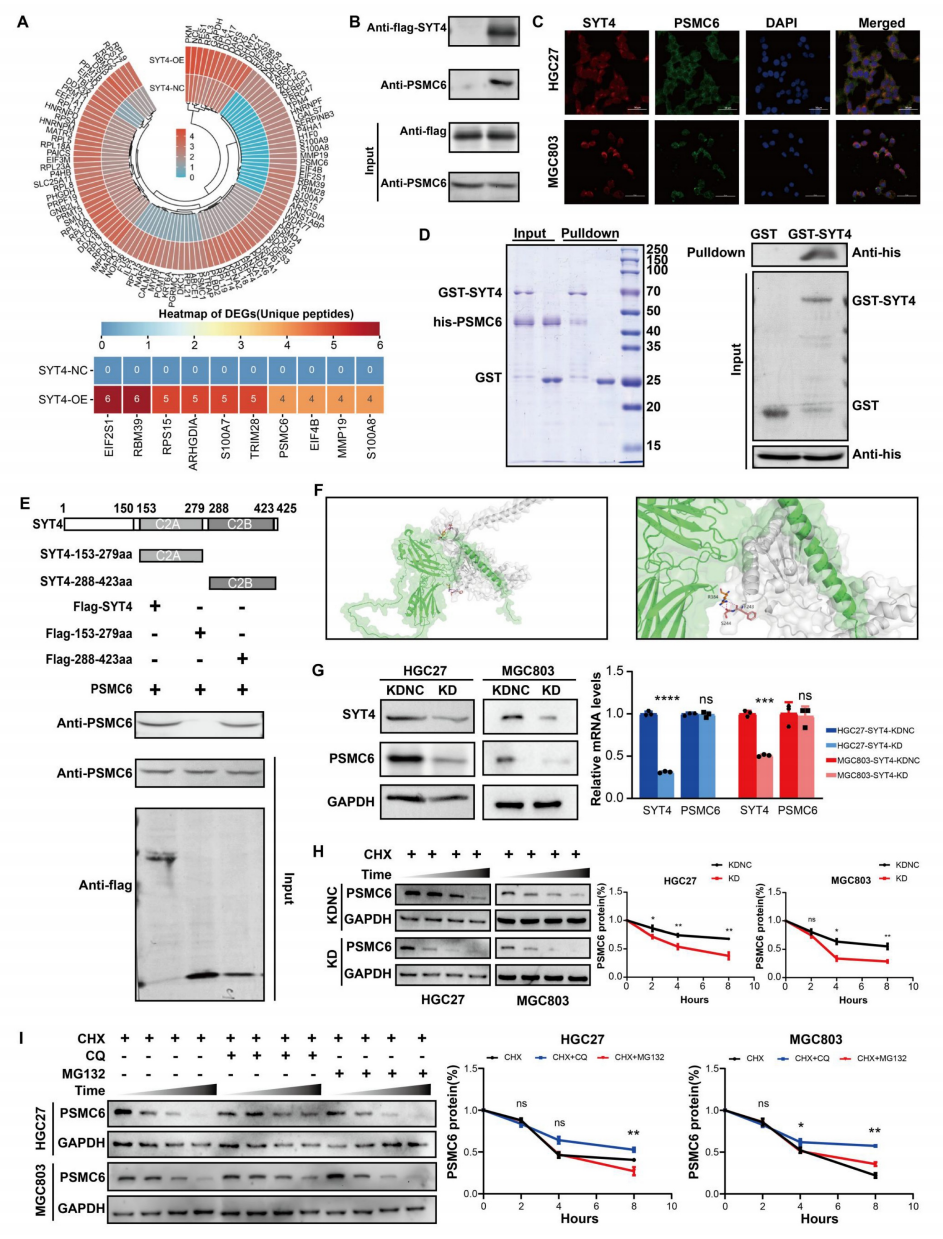
** SYT4 directly interacts with PSMC6. A** total of 106 potential binding proteins for SYT4 were discovered by IP-MS. **B** Co-IP detected that SYT4 interacts with the protein PSMC6. **C** SYT4 and PSMC6 were co-localized in cytoplasm by immunofluorescence. **D** GST pull-down verified the direct interaction of SYT4 and PSMC6. **E** and **F** The C2B domain (288-423 aa) of SYT4 was essential for interaction with PSMC6. **G** Knockdown of SYT4 downregulated PSMC6 protein level, but not transcript level. **H** SYT4 knockdown markedly decreased the half-life of PSMC6 protein. **I** SYT4 stabilized PSMC6 protein by autophagy-lysosome pathway.

To clarify how SYT4 drives GC progression, we employed co-immunoprecipitation (co-IP) coupled with immunoprecipitation-mass spectrometry (IP-MS) to screen for SYT4-binding proteins. By comparing peptide counts between SYT4-overexpressing cells and control cells, we identified potential interacting proteins. A protein was deemed significant if its peptide count was at least double that in the control, with a minimum difference of four peptides. Detailed findings are in Supplementary Table S6. This approach uncovered 106 potential SYT4-binding partners (Fig. 4A). Among the top 10 proteins in the differential ranking, we noticed a protein, PSMC6, linked to the Wnt/β-catenin pathway (Fig. 4A). Co-IP assays confirmed the interaction between SYT4 and PSMC6 (Fig. 4B), and immunofluorescence staining also confirmed the co-localization of SYT4 and PSMC6 proteins in the cytoplasm (Fig. 4C). Subsequently, GST pull-down assays further validated this direct interaction (Fig. 4D). To identify the specific region of SYT4 that interacts with PSMC6, we generated full-length SYT4 and two truncated mutants. Only the SYT4-C2B mutant (amino acids 288-423) co-precipitated with PSMC6 (Fig. 4E). Molecular docking predicted that PSMC6 primarily interacts with SYT4's second C2 domain (C2B, amino acids 288-423). Specifically, the Arg-384 residue of SYT4 forms hydrogen bonds with the Ser-244 and Phe-243 residues of PSMC6 (Fig. 4F).

Knockdown of SYT4 downregulated protein level, but not transcript level of PSMC6 in GC cells (Fig. 4G). Therefore, we further verified whether SYT4 could modulate the stability of PSMC6 protein. Cells were pre-treated with CHX, as shown in Figure 4H, silencing of SYT4 could significantly shortened the half-life of PSMC6 protein. To explore the degradation pathway, cells were co-treated GC cell with CHX and MG-132 (10 µM, proteasome inhibitor) or chloroquine (CQ, 25 µM, lysosome inhibitor). Notably, incubated with CQ attenuated the degradation of PSMC6 protein (Figure 4I). Collectively, these results indicated that SYT4 could stabilize PSMC6 protein via autophagy-lysosome pathway.

To examine the correlation between SYT4 and PSMC6 expression in GC patients, immunohistochemistry (IHC) staining for PSMC6 was performed on tissue microarrays. Results showed that PSMC6 expression was significantly higher in GC tissues (42.33% of 1429 samples) than in adjacent non-cancerous tissues (10.4% of 1105 samples, *P* < 0.001, Supplementary Figure S4A). Pearson correlation analysis also indicated a positive correlation between SYT4 and PSMC6 expression in GC tissues (R = 0.560, *P* < 0.001, Supplementary Figure S4B).

### The oncogenic role of SYT4 and activation of the Wnt/β-catenin pathway depend on PSMC6

**Figure 5:**
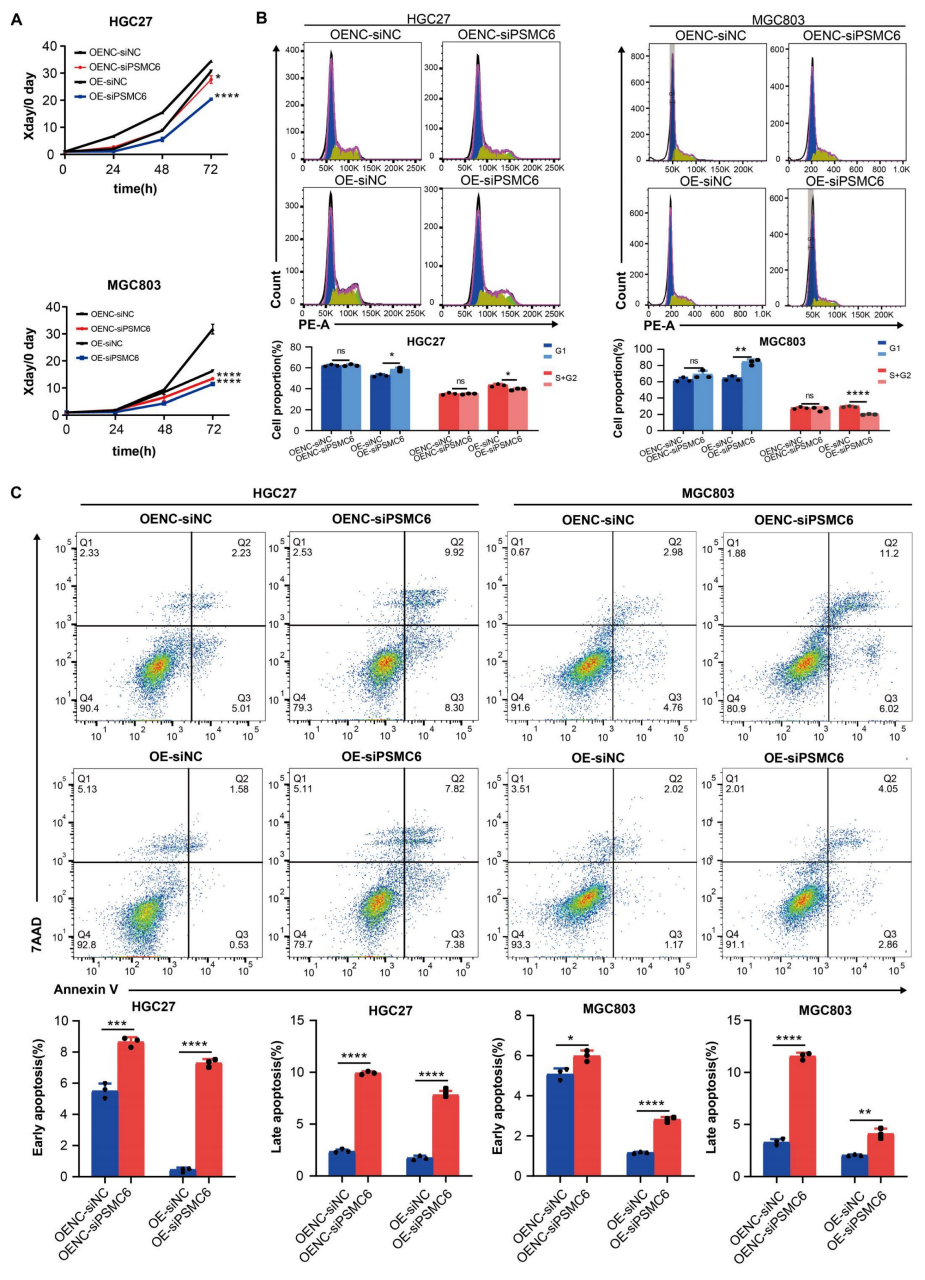
** The oncogenic role of SYT4 was partially dependent on PSMC6. A** Knockdown of PSMC6 significantly abrogated the promoting effects of SYT4 on cell proliferation. **B** Knockdown of PSMC6 reversed the cell cycle progression caused by SYT4 overexpression. **C** Knockdown of PSMC6 markedly reversed cell apoptosis induced by SYT4.

To assess PSMC6's role in SYT4-driven gastric cancer progression, we co-transfected PSMC6 knockdown plasmids into SYT4-overexpressing (OE) cells. CCK8 assays revealed that PSMC6 knockdown significantly reversed SYT4's effects on cell proliferation (Fig. 5A), cell cycle progression (Fig. 5B), and apoptosis (Fig. 5C) in both HGC27 and MGC803 cells.

**Figure 6:**
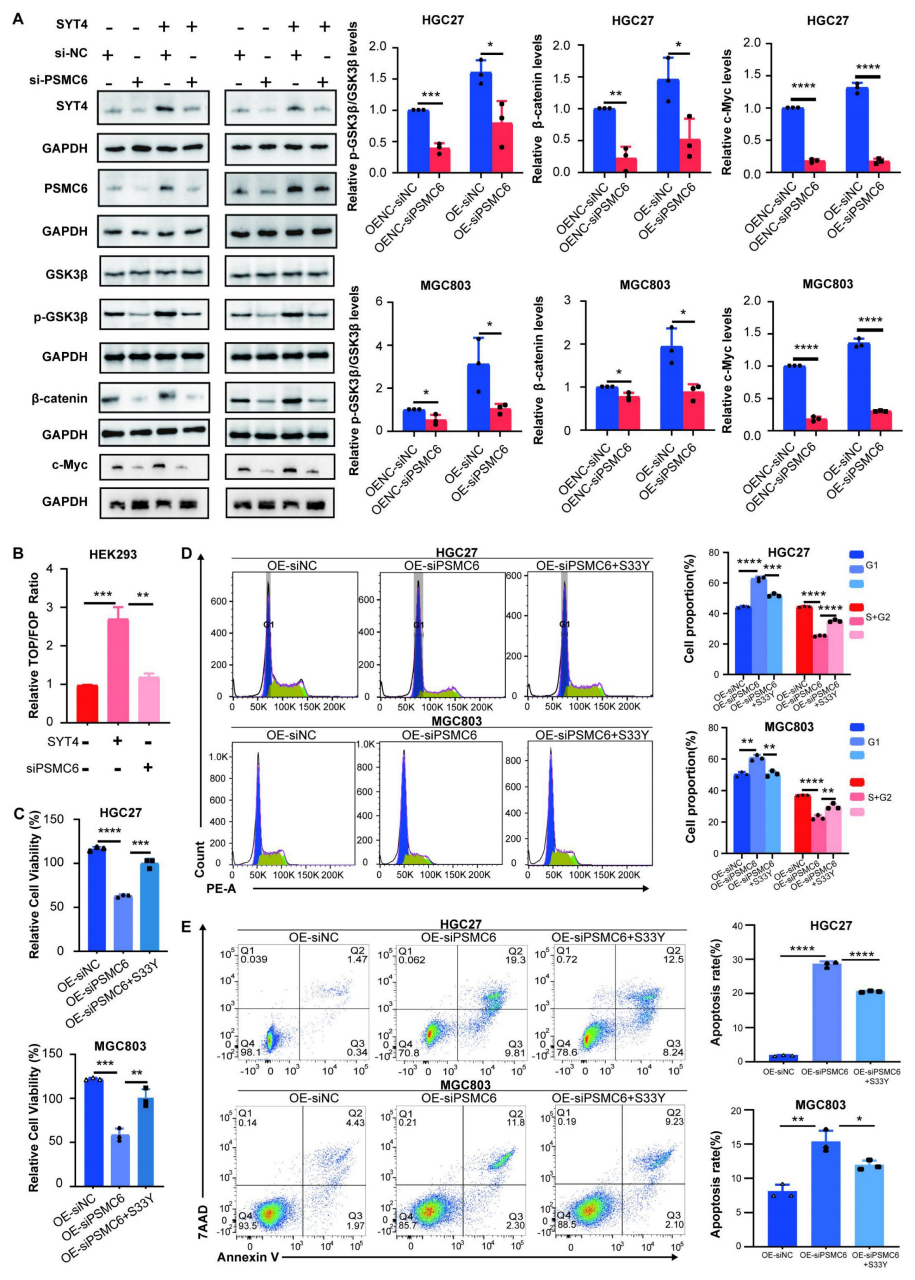
** The SYT4 and PSMC6 complex plays an oncogenic role in GC by regulating Wnt/β-catenin signaling. A** The upregulated expression of p-GSK3β/GSK3β and β-catenin was significantly downregulated by attenuation of PSMC6. **B** PSMC6 knockdown partially abrogated the SYT4-mediated activation of the Wnt/β-catenin pathway, as evidenced by a dual luciferase reporter assay. **C** The inhibitory effects on cell proliferation were reversed in SYT4-OE-siPSMC6 cells transfected with a constitutively active β-catenin mutant (S33Y). **D** and **E** The effects on cell cycle progression and apoptosis were rescued when β-catenin_S33Y_ was overexpressed.

We then investigated whether PSMC6 is involved in the SYT4-regulated Wnt/β-catenin pathway. As expected, PSMC6 knockdown led to a notable reduction in p-GSK3β/GSK3β and β-catenin protein levels (Fig. 6A). The TOP/FOP-Flash reporter assay showed that PSMC6 silencing inhibited the activation of the Wnt/β-catenin pathway induced by SYT4 overexpression (Fig. 6B). To determine if the oncogenic effects of the SYT4-PSMC6 interaction depend on β-catenin activation, we transfected SYT4-OE-siPSMC6 cells with a constitutively active β-catenin^S33Y^ mutant. In SYT4-OE cells, PSMC6 downregulation suppressed proliferation and arrested cells in the G1 phase, but these effects were reversed by β-catenin^S33Y^ overexpression (Fig. 6C, 6D). Moreover, β-catenin^S33Y^ reduced the high apoptosis rate observed in SYT4-OE-siPSMC6 cells (Fig. 6E). These results demonstrated that SYT4's oncogenic effects and its activation of the Wnt/β-catenin pathway partially rely on PSMC6.

### Identification of potential SYT4 inhibitors via compound screening

**Figure 7:**
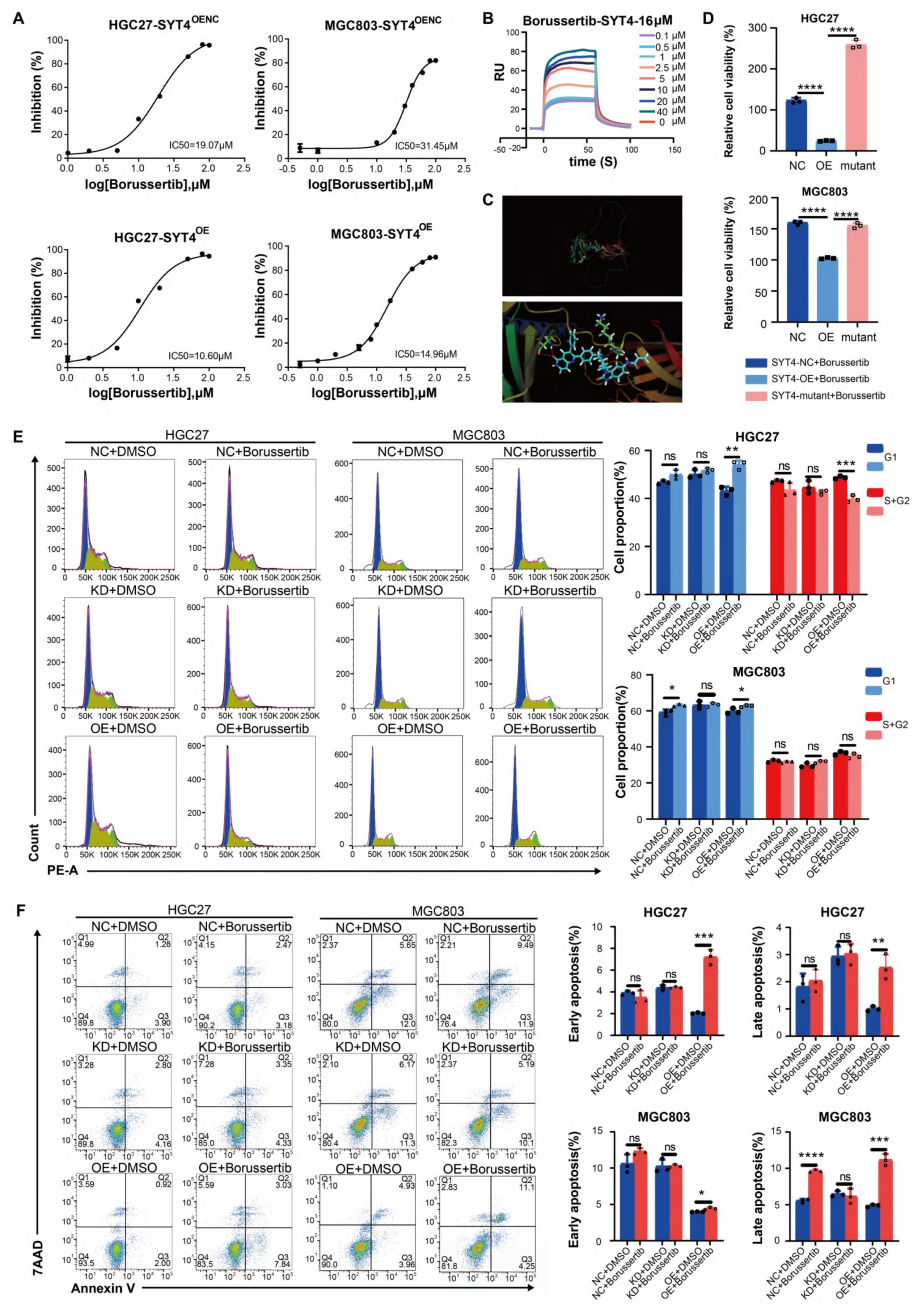
** Borussertib specifically affected SYT4-induced cell viability, cell cycle and apoptosis. A** Borussertib was more sensitive at a lower concentration in the SYT4 overexpression groups than in the control groups among HGC27 and MGC803 cells. **B** The SPR method showed the affinity KD value for borussertib with SYT4 protein. **C** Borussertib formed a unique covalent bond with SYT4 by 4 hydrogen bonds at Asp251, Lys284 and Ser285. **D** The attenuation of inhibition effect of borussertib in SYT4-mutant group. **E** and **F** Borussertib induced cell cycle arrest in the G1 phases and blocked the effect of SYT4 on apoptosis in the SYT4 overexpression groups but not in the control and SYT4 knockdown groups.

In our pursuit of SYT4 inhibition as a therapeutic strategy for GC, we screened a library of 517 compounds to identify SYT4 inhibitors. These compounds were tested on SYT4-overexpressing (OE) cells and control cells (SYT4-OENC). Among them, 76 compounds demonstrated significantly stronger inhibitory effects in the SYT4-OE group compared to the control group (*P* < 0.05). Borussertib emerged as the most potent inhibitor, exhibiting a 2-fold higher inhibition in SYT4-OE cells. Regression analysis revealed that the half-maximal inhibitory concentration (IC50) of borussertib in HGC27 cells was 19.07 μM for SYT4-OENC and 10.60 μM for the SYT4-OE group. Similarly, in MGC803 cells, the IC50 values were 31.45 μM for SYT4-OENC and 14.96 μM for the SYT4-OE group. These results indicated that borussertib is more effective at lower concentrations in the SYT4-OE group compared to the control group (Fig. 7A).

To investigate whether the enhanced inhibitory effect of borussertib in SYT4-OE cells was due to specific binding between the compound and SYT4, we employed surface plasmon resonance (SPR). SPR analysis confirmed that borussertib binds to SYT4 with a dissociation constant (KD) of 16 µM (Fig. 7B). Furthermore, docking studies revealed that borussertib forms a unique covalent bond with SYT4 through four hydrogen bonds at the Asp251, Lys284, and Ser285 residues (Fig. 7C).

### Borussertib reverses SYT4-mediated proliferation in GC

The potential of SYT4 as a drug target in GC was further confirmed by mutating the Borussertib-SYT4 specific binding sites Asp251, Lys284, and Ser285 to alanine, generating a SYT4-mutant (Supplementary Figure S5). Upon treatment with 15 μM borussertib—a concentration determined based on the IC50 and binding affinity KD value—the inhibitory effect was significantly reduced in the SYT4-mutant group compared to the SYT4-overexpressing (OE) groups (Fig. 7D). This result ruled out the possibility of off-target effects of borussertib on SYT4.

To investigate the effects of borussertib on GC cells, we treated control, SYT4-knockdown (KD), and SYT4-OE groups with the drug. Borussertib alleviated cell cycle arrest in the G1 and S/G2 phases in the SYT4-OE group, while showing minimal effects on the control and SYT4-KD groups (Fig. 7E). Regarding apoptosis, borussertib had no significant impact on the control and SYT4-KD groups but markedly promoted apoptosis in the SYT4-OE group, thereby demonstrating its specificity in targeting SYT4 (Fig. 7F).

### Effect of borussertib on xenograft models

**Figure 8:**
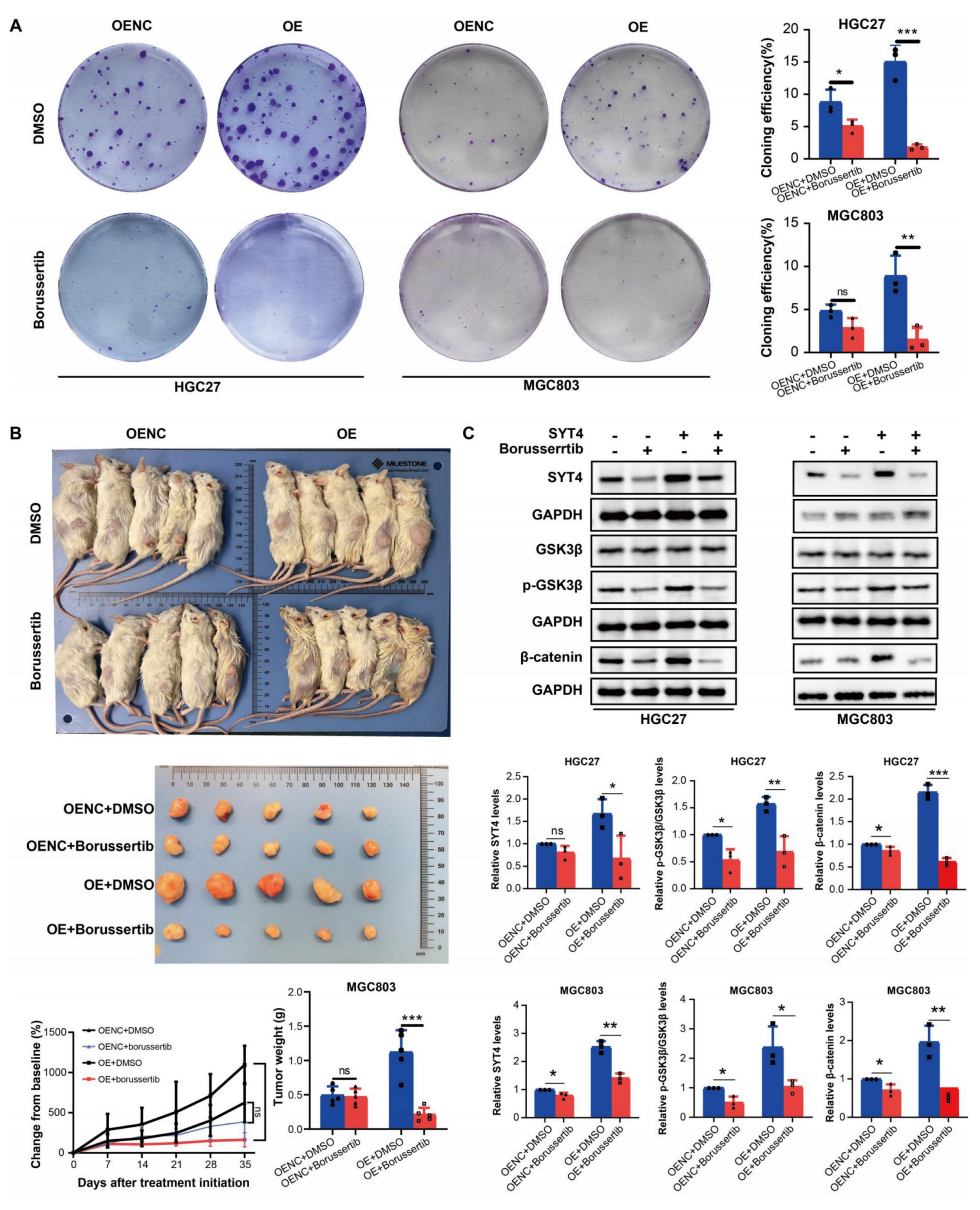
** Borussertib attenuated the tumor-promoting function of SYT4 *in vitro* and *in vivo***.** A** Compared with SYT4 control groups, SYT4 overexpression groups exhibited a significant reduction in colony formation after borussertib therapy. **B** The *in vivo* effect of borussertib in the SYT4-mediated xenograft model. **C** The expressions of SYT4, p-GSK3β/GSK3β and β-catenin was determined in the SYT4-control, SYT4-control+borussertib, SYT4-OE and SYT4-OE+borussertib groups.

To assess the antitumor potential of borussertib *in vivo*, we evaluated its effects on xenograft models. Borussertib significantly suppressed colony formation in MGC803 cells with both SYT4 overexpression (OE) and normal expression (OENC), with the most pronounced inhibitory effect observed in the SYT4-OE group (Fig. 8A). *In vivo* studies showed that borussertib, administered at a dose of 20 mg/kg, effectively inhibited tumor growth in both MGC803-SYT4-OE and MGC803-SYT4-OENC xenograft models. Notably, the SYT4-OE group demonstrated greater sensitivity to borussertib compared to the OENC group (Fig. 8B), a finding consistent with the colony formation assay results. These data highlight SYT4 as a promising therapeutic target for GC, suggesting that borussertib could effectively suppress GC tumor growth both *in vitro* and *in vivo*.

### Borussertib suppresses SYT4-mediated Wnt/β-catenin signaling

The effect of borussertib on SYT4 expression and Wnt target genes was assessed. The drug markedly lowered SYT4 levels in the SYT4-overexpressing (OE) group, with minimal changes in the control (OENC) groups (Fig. 8C). Moreover, Borussertib more effectively reduced p-GSK3 β/GSK3 β and β-catenin levels in SYT4-OE cells than in OENC cells, indicating that it disrupts the SYT4-mediated Wnt/β-catenin pathway in GC (Fig. 8C).

## Discussion

Gastric cancer (GC), a prevalent malignant tumor of the digestive tract, arises from a complex pathophysiological process involving multiple gene mutations and evolutionary features 17. Despite advancements in treatments based on GC's molecular pathology, clinical outcomes remain suboptimal, largely due to many molecular markers not yet being therapeutically leveraged or fully elucidated in terms of their precise mechanisms 18. Thus, identifying effective biomarkers and exploring their underlying molecular mechanisms warrant further study. Increasing studies have shown an association between abnormal gene expression of GC-related genes and malignant prognosis, as well as progression during GC 19, 20.

SYT4 plays a crucial role in regulating calcium-dependent vesicle fusion and enhancing presynaptic function 21. Recent studies have linked SYT family members to human cancers 22, 23. However, the specific importance of SYT4 in GC has not been well understood. In this study, we analyzed the differential proteome data from 90 GC samples using bioinformatics methods and identified SYT4 as a potential prognostic marker in GC. Microarray results showed that SYT4 expression is elevated in GC tissue. High SYT4 levels were associated with several factors, including increased Ki-67 expression, lymph node metastasis, poor tissue differentiation, advanced T stage, perineural invasion, vascular invasion, TNM stage, and larger tumor size. Patients with higher SYT4 expression had poorer prognoses.

Subsequent experiments demonstrated that knocking down SYT4 significantly reduced GC cell growth, halted the G1-S phase transition, and promoted cell apoptosis. Conversely, SYT4 overexpression had opposite effects. In a mouse model with subcutaneous xenografts, SYT4 knockdown also led to reduced tumor growth, consistent with our *in vitro* findings. These results strongly suggest that SYT4 may play a critical role in the development of GC. Studies on the signal transduction underlying the function of SYT4 have been reported. For instance, during melanogenesis, SYT4 participates in regulating Ca^2+^ influx via the TRPM1 channel 9. Mori et al. 24 indicated that nerve growth factor induces JNK-mediated SYT4 phosphorylation. Nevertheless, the precise mechanism by which SYT4 facilitates GC progression has not been fully elucidated. Based on the results of GSEA and protein array analysis, we found that the Wnt pathway might be the potential signaling pathway mediated by SYT4. The Wnt/β-catenin signaling cascade is integral to gastric tumorigenesis, regulating cell proliferation, differentiation, and apoptosis 25. The regulation of the transcription coactivator β-catenin is maintained by the β-catenin destruction complex 26. In the Wnt-on state, GSK3β phosphorylation inhibits β-catenin degradation, leading to its stabilization and translocation to the nucleus. This process facilitates the activation of downstream genes, such as c-Myc, vimentin, and MMPs, via T cell factor/lymphoid enhancer factor (TCF/LEF) transcription factors 27. As a target gene of β-catenin, c-Myc regulates cell proliferation, apoptosis, differentiation, and metabolism 28. In this study, SYT4 overexpression increased GSK3β phosphorylation and β-catenin levels, while SYT4 knockdown had the opposite effect. SYT4 upregulation also promoted the nuclear translocation of β-catenin, ultimately increasing c-Myc expression. Thus, we hypothesize that SYT4 regulates the Wnt pathway via its downstream effector, β-catenin.

To better understand how SYT4 modulates the Wnt/β-catenin pathway, we performed co-immunoprecipitation (Co-IP) followed by immunoprecipitation-mass spectrometry (IP-MS) to identify interacting proteins of SYT4. PSMC6, located in the basal region of the proteasome 19S regulatory particle and functioning as an ATPase 29, was identified as a key interacting protein. PSMC6 dysfunction has been confirmed in many cancers 30, and interfering with PSMC6 expression can suppress cell proliferation and promote apoptosis 31. Zhang et al. 31 reported that PSMC6 upregulation activates the Wnt signaling pathway by degrading the AXIN complex. Herein, we verified the direct interaction between SYT4 and PSMC6, which could stabilize the expression of PSMC6 protein. Analysis of SYT4 truncated mutants and protein-protein interaction predictions revealed that only full-length SYT4 and its C2B domain (288-423 aa) could co-precipitate with PSMC6, indicating that the interaction between SYT4 and PSMC6 is dependent on SYT4's protein structure and requires further exploration. When PSMC6 levels were reduced, it decreased cell proliferation, blocked cell cycle progression, and promoted apoptosis induced by SYT4. It also inhibited the activation of Wnt/β-catenin signaling. To further investigate this, we introduced a mutated form of β-catenin (S33Y), which is resistant to degradation, into SYT4-overexpressing cells with reduced PSMC6 levels 32. The results demonstrated that S33Y could reverse the effects on cell growth, cell cycle distribution, and apoptosis. Based on these findings, we infer that SYT4 and PSMC6 cooperatively regulate GC progression by targeting β-catenin.

In clinical settings, the effectiveness of targeted treatments for GC patients remains less than ideal. Identifying precise targets and effective drugs could pave the way for personalized treatments. During the screening of various compounds, borussertib was identified as a highly selective inhibitor that effectively suppresses the proliferation of GC cell lines driven by SYT4. Borussertib, known as an irreversible allosteric inhibitor of the protein AKT, has demonstrated its ability to inhibit cell proliferation in preliminary studies involving colon and pancreatic cancers with KRAS mutations 11, 33. In this study, we mutated the borussertib binding sites on SYT4 to disrupt the specific drug-protein binding and observed a significant reduction in the inhibitory effect, thereby ruling out the off-target effects of borussertib. Additionally, borussertib reduced SYT4-induced functionalities in GC cells, although this effect was limited in SYT4-NC and SYT4-KD cells. These results indicate that borussertib targets SYT4-mediated GC. This hypothesis was supported in xenograft models, where borussertib significantly reduced tumor growth in the SYT4-OE model. Furthermore, we investigated whether borussertib affects the SYT4-mediated upregulation of β-catenin. We discovered that borussertib treatment triggered a downregulation of SYT4 expression and suppressed the levels of p-GSK3β/GSK3β and β-catenin. Therefore, borussertib exhibited an on-target effect on SYT4 and shows promising potential to reduce SYT4-mediated signal transduction, providing valuable insights into the therapeutic potential of targeting SYT4 in GC.

## Conclusions

The present investigation establishes a clinical and mechanistic basis for SYT4 as a novel biomarker in GC, underscoring its critical role in tumorigenesis. The oncogenic effects of SYT4 are attributed to its direct interaction with and stabilization of PSMC6 protein, which subsequently activates the Wnt/β-catenin pathway. Our findings indicate that borussertib, a selective inhibitor, could be developed into a targeted therapy to specifically disrupt the cancer-promoting actions of SYT4. This offers a promising and effective approach for GC treatment. By elucidating the molecular mechanisms of SYT4 and demonstrating the efficacy of borussertib, our study lays the groundwork for further research and development of targeted therapies for GC. This work also opens up possibilities for personalized medicine, where treatments can be tailored to the specific molecular characteristics of a patient's tumor, potentially leading to improved outcomes for GC patients.

## Supplementary Material

Supplementary figures and tables 1-3.

Supplementary table 4.

Supplementary table 5.

Supplementary table 6.

## Figures and Tables

**Table 1:** Correlation of clinicopathological features of SYT4 in 1429 patients

	[ALL]*N=1429*	None*N=556*	Low*N=285*	High*N=588*	p.overall
**SYT4.Hscore**	20.00 [0.00;40.00]	0.00 [0.00;0.00]	20.00 [10.00;20.00]	60.00 [30.00;60.00]	**< 0.001**
**Gender**					**0.028**
Male	1044 (73.06%)	385 (69.24%)	211 (74.04%)	448 (76.19%)	
Female	385 (26.94%)	171 (30.76%)	74 (25.96%)	140 (23.81%)	
**Age (years)**	63.00 [56.00;70.00]	62.00 [54.00;68.00]	64.00 [58.00;71.00]	64.00 [57.00;71.00]	**< 0.001**
**Her2 status**					**< 0.001**
Negative	417 (29.18%)	193 (34.71%)	88 (30.88%)	136 (23.13%)	
Low-expression	885 (61.93%)	334 (60.07%)	177 (62.11%)	374 (63.61%)	
Amplification	127 (8.89%)	29 (5.22%)	20 (7.02%)	78 (13.27%)	
**Epstein-Barr virus infection**					**0.001**
No	1241 (86.84%)	505 (90.83%)	246 (86.32%)	490 (83.33%)	
Yes	188 (13.16%)	51 (9.17%)	39 (13.68%)	98 (16.67%)	
**Lymphatic metastasis**					**0.001**
No	448 (31.35%)	206 (37.05%)	77 (27.02%)	165 (28.06%)	
Yes	981 (68.65%)	350 (62.95%)	208 (72.98%)	423 (71.94%)	
**N stage**					**< 0.001**
0	425 (29.74%)	295 (53.07%)	73 (25.61%)	57 (26.70%)	
1	256 (17.91%)	96 (17.27%)	52 (18.25%)	108 (18.37%)	
2	318 (22.26%)	111 (19.96%)	65 (22.81%)	142 (24.15%)	
3a	239 (16.73%)	94 (16.91%)	61 (21.40%)	84 (14.29%)	
3b	191 (13.37%)	60 (10.79%)	34 (11.93%)	96 (16.33%)	
**Deposi**t					**0.003**
No	1118 (78.24%)	461 (82.91%)	212 (74.39%)	445 (75.68%)	
Yes	311 (21.76%)	95 (17.09%)	73 (25.61%)	143 (24.32%)	
**Differentiation**					**< 0.001**
Well-differentiation	1 (0.07%)	0 (0.00%)	1 (0.35%)	0 (0.00%)	
Moderate-differentiation	291 (20.36%)	100 (17.99%)	37 (12.98%)	154 (26.19%)	
Poor-differentiation	1137 (79.57%)	456 (82.01%)	248 (86.67%)	433 (73.64%)	
**T stage**					**< 0.001**
1	247 (17.28%)	157 (28.24%)	31 (10.88%)	59 (9.52%)	
2	226 (15.82%)	96 (17.27%)	39 (13.68%)	91 (15.48%)	
3	357 (24.98%)	114 (20.50%)	67 (23.86%)	175 (29.76%)	
4	599 (41.92%)	189 (33.99%)	148 (51.58%)	262 (45.24%)	
**Nerve invasion**					**0.001**
No	640 (44.79%)	278 (50.00%)	104 (36.49%)	258 (43.88%)	
Yes	789 (55.21%)	278 (50.00%)	181 (63.51%)	330 (56.12%)	
**Vascular invasion**					**< 0.001**
No	645 (45.14%)	298 (53.60%)	103 (36.14%)	244 (41.50%)	
Yes	784 (54.86%)	258 (46.40%)	182 (63.86%)	344 (58.50%)	
**pTNM stage**					**< 0.001**
1	298 (20.85%)	168 (30.22%)	46 (16.14%)	84 (14.29%)	
2	352 (24.62%)	119 (21.40%)	61 (21.40%)	174 (29.59%)	
3	747 (52.27%)	255 (45.86%)	171 (60.00%)	321 (54.59%)	
4	32 (2.24%)	14 (2.52%)	7 (2.46%)	11 (1.87%)	
**Tumor size (cm)**	4.00 [2.50;5.50]	3.50 [2.00;5.00]	4.00 [3.00;6.00]	4.00 [3.00;6.00]	**< 0.001**
**Lauren classification**					**< 0.001**
Uncertain	65 (4.55%)	10 (1.80%)	16 (5.61%)	39 (6.63%)	
Intestinal	354 (24.77%)	106 (19.06%)	51 (17.89%)	197 (33.50%)	
Mixed	648 (45.35%)	248 (44.60%)	135 (47.37%)	265 (45.07%)	
Diffuse	362 (25.33%)	192 (34.53%)	83 (29.12%)	87 (14.80%)	

## Abbreviations

GC: Gastric cancer
SYT4: Synaptotagmin-4
IP-MS: Immunoprecipitation-mass spectrometry
Co-IP: Co-immunoprecipitation
PSMC6: Proteasome 26S subunit ATPase 6
FFPE: Formalin-fixed, paraffin-embedded
RF: Random forest
IHC: Immunohistochemistry
qRT‒PCR: Quantitative real-time transcription PCR
WB: western blotting
PMSF: phenylmethylsulfonyl fluoride
NC: Negative control
KD: Knockdown
OE: Overexpressiong
CHX: Cycloheximide
CQ: Chloroquine
CCK-8: Cell counting assay Kit-8
IC50: Half-maximal inhibitory concentration
SPR: Surface plasmon resonance
iBAQ: Intensity-based absolute quantification
OS: Overall survival
DSS: Disease-specific survival
PFS: Progression-free survival
PFI: Progression-free interval
GSEA: Gene Set Enrichment Analysis
TCF/LEF: T cell factor/lymphoid enhancer factor
