# Supplementary material for: SYT4 Interacts with PSMC6 to Facilitate Malignant Progression in Gastric Carcinoma via Activating Wnt/β-catenin Signaling
Source: Int J Biol Sci. 2025 Oct 20;21(15):6775–93. doi: 10.7150/ijbs.118672 (PMC12631102; doi:10.7150/ijbs.118672)
Supplement: Supplementary file 1 — Supplementary figures and tables 1-3. [file ijbsv21p6775s1.pdf]

Supplementary Information

Fig. S1. COX regression analysis of FKBP9 in GC.

Fig. S2. The expression of SYT4 in different cells. A The mRNA levels and protein levels of SYT4 in GES1, AGS, HGC27, BGC823, MKN45 and MGC803 cells were measured by qRT–PCR and WB. **B** The transduction efficiency of SYT4 overexpression and knockdown in HGC27 and MGC803 cells.

Fig. S3. Effect of SYT4 on the invasion and migration ability of GC cells. A and **B** Transwell and scratch assays showed the inefficiency of SYT4 overexpression on the invasion and migration in HGC27 and MGC803 cells.

Fig. S4. Correlation between SYT4 and PSMC6 expression in GC. A Representative images of SYT4 and PSMC6 staining in GC. **B** Spearman analysis showed the expression of SYT4 and PSMC6 was positively correlated.

Fig. S5. The transfection efficiency of SYT4-mutant lentivirus.

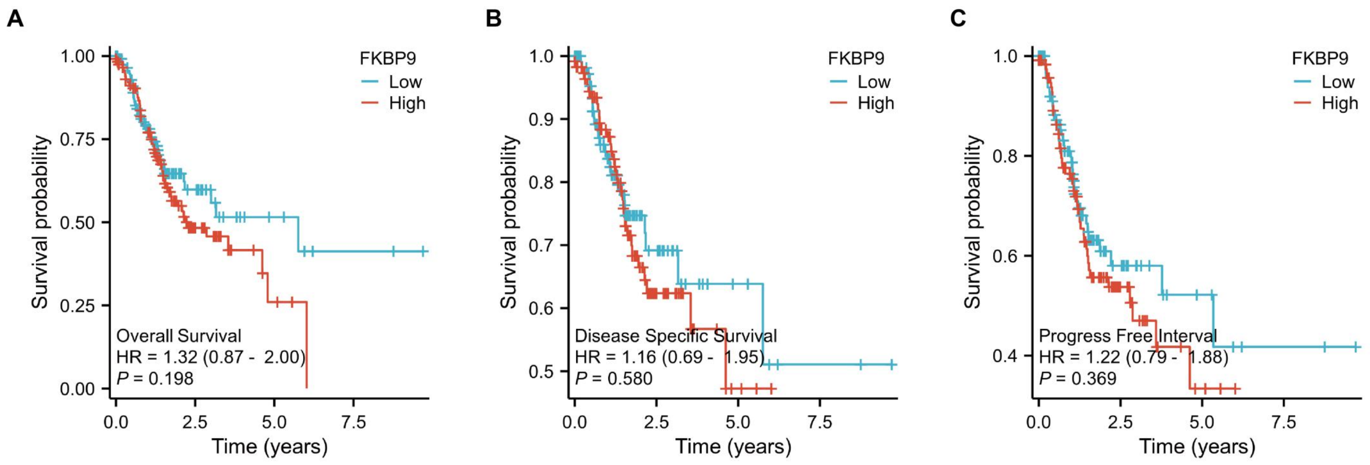
A
B
C
FKBP9
Low
High
Survival probability
Time (years)
Overall Survival
HR = 1.32 (0.87 - 2.00)
P = 0.198
Disease Specific Survival
HR = 1.16 (0.69 - 1.95)
P = 0.580
Progress Free Interval
HR = 1.22 (0.79 - 1.88)
P = 0.369

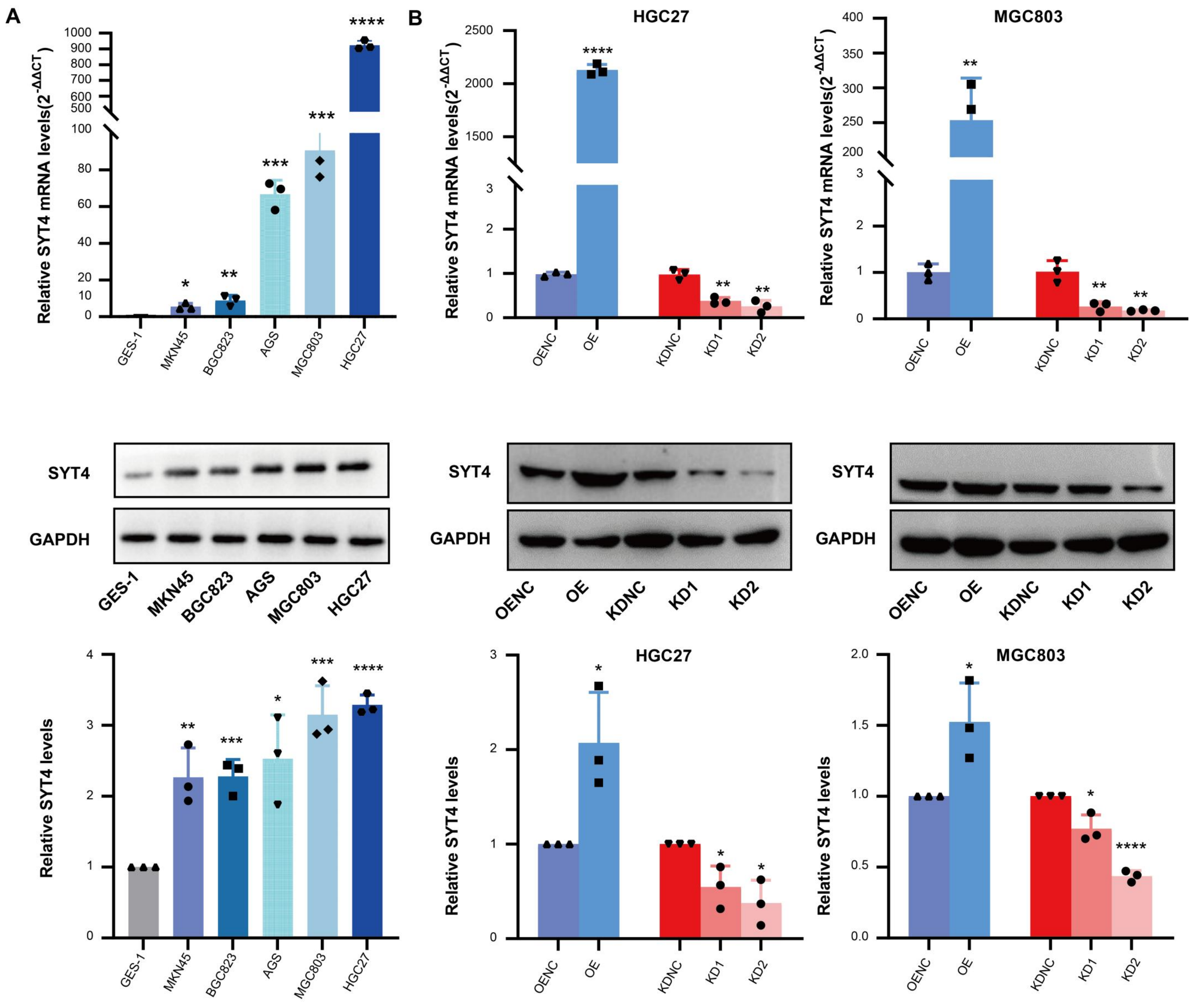

A
B
HGC27
MGC803
Relative SYT4 mRNA levels(2^-ΔΔCT)
GES-1
MKN45
BGC823
AGS
MGC803
HGC27
OENC
OE
KDNC
KD1
KD2
SYT4
GAPDH
Relative SYT4 levels

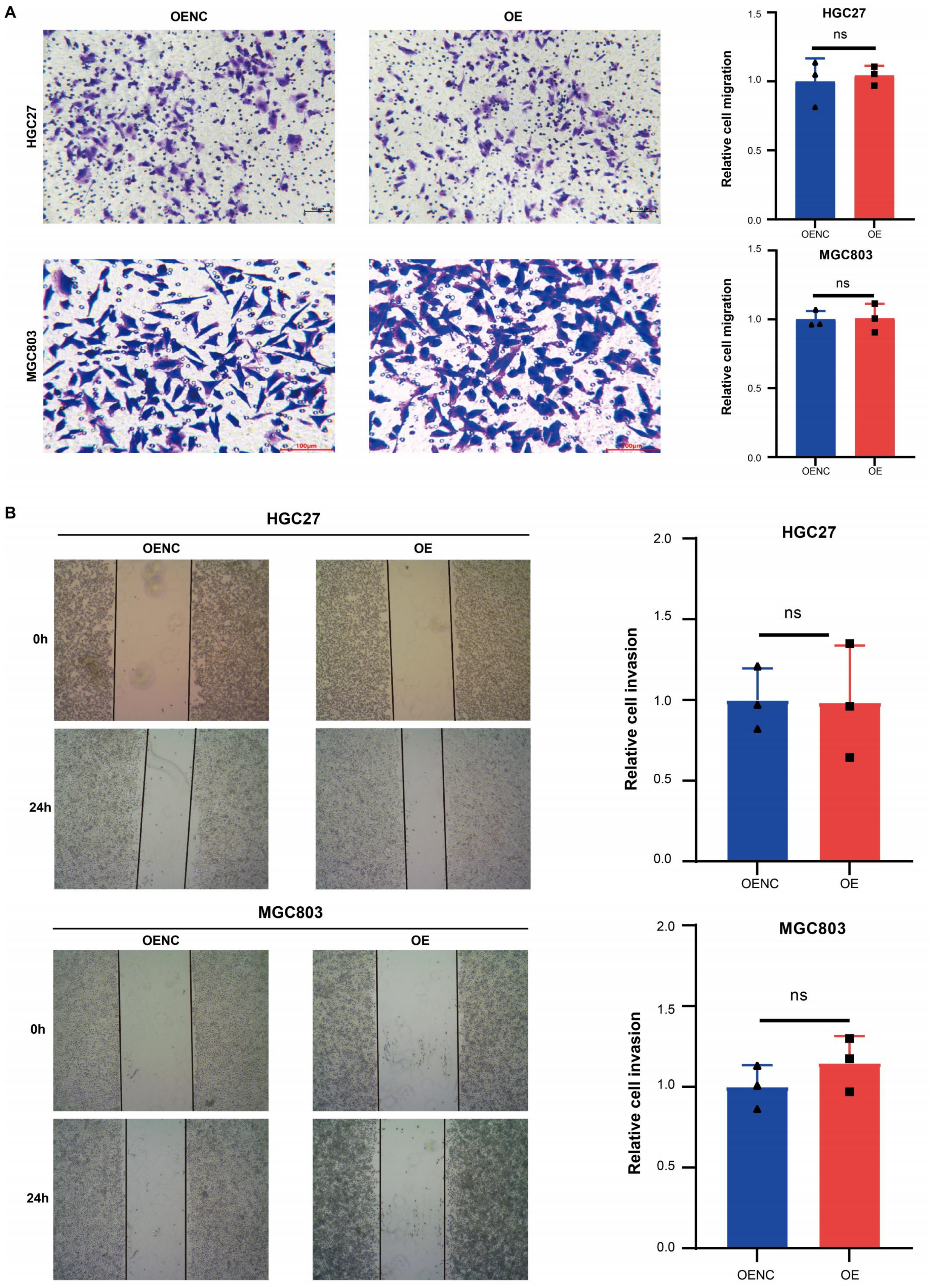
A
OENC
OE
HGC27
MGC803
100μm
HGC27
MGC803
ns
Relative cell migration
1.5
1.0
0.5
0.0
OENC
OE
B
HGC27
OENC
OE
0h
24h
MGC803
OENC
OE
0h
24h
HGC27
MGC803
ns
Relative cell invasion
2.0
1.5
1.0
0.5
0.0
OENC
OE

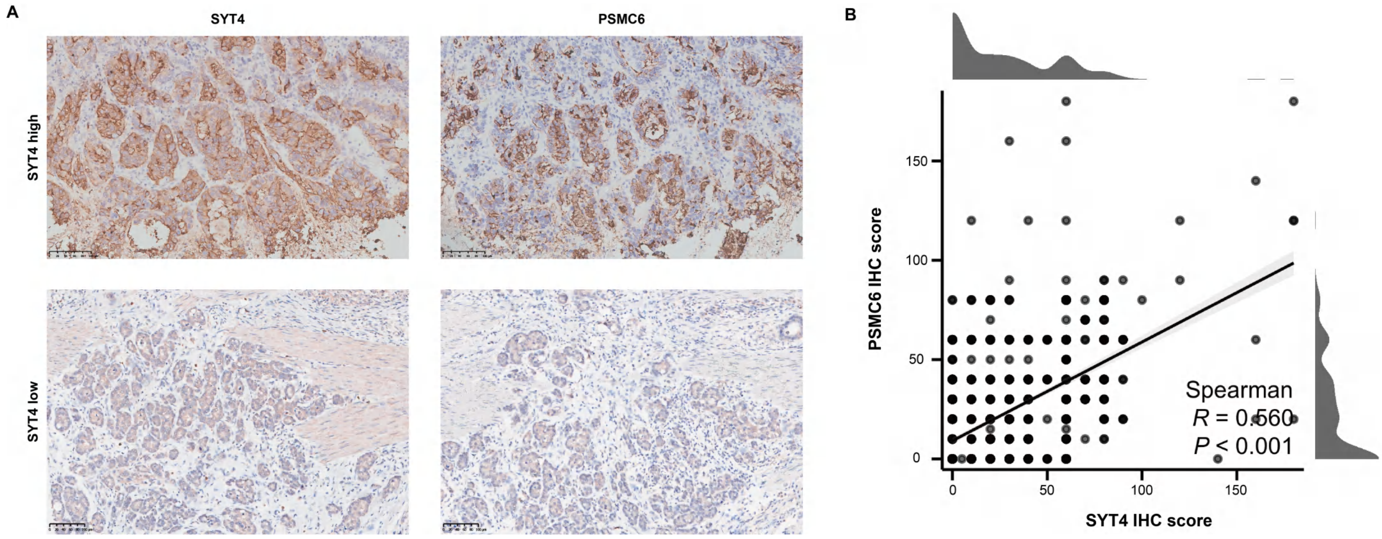
A
SYT4
PSMC6
SYT4 high
SYT4 low
B
PSMC6 IHC score
SYT4 IHC score
Spearman
R = 0.560
P < 0.001
0
50
100
150

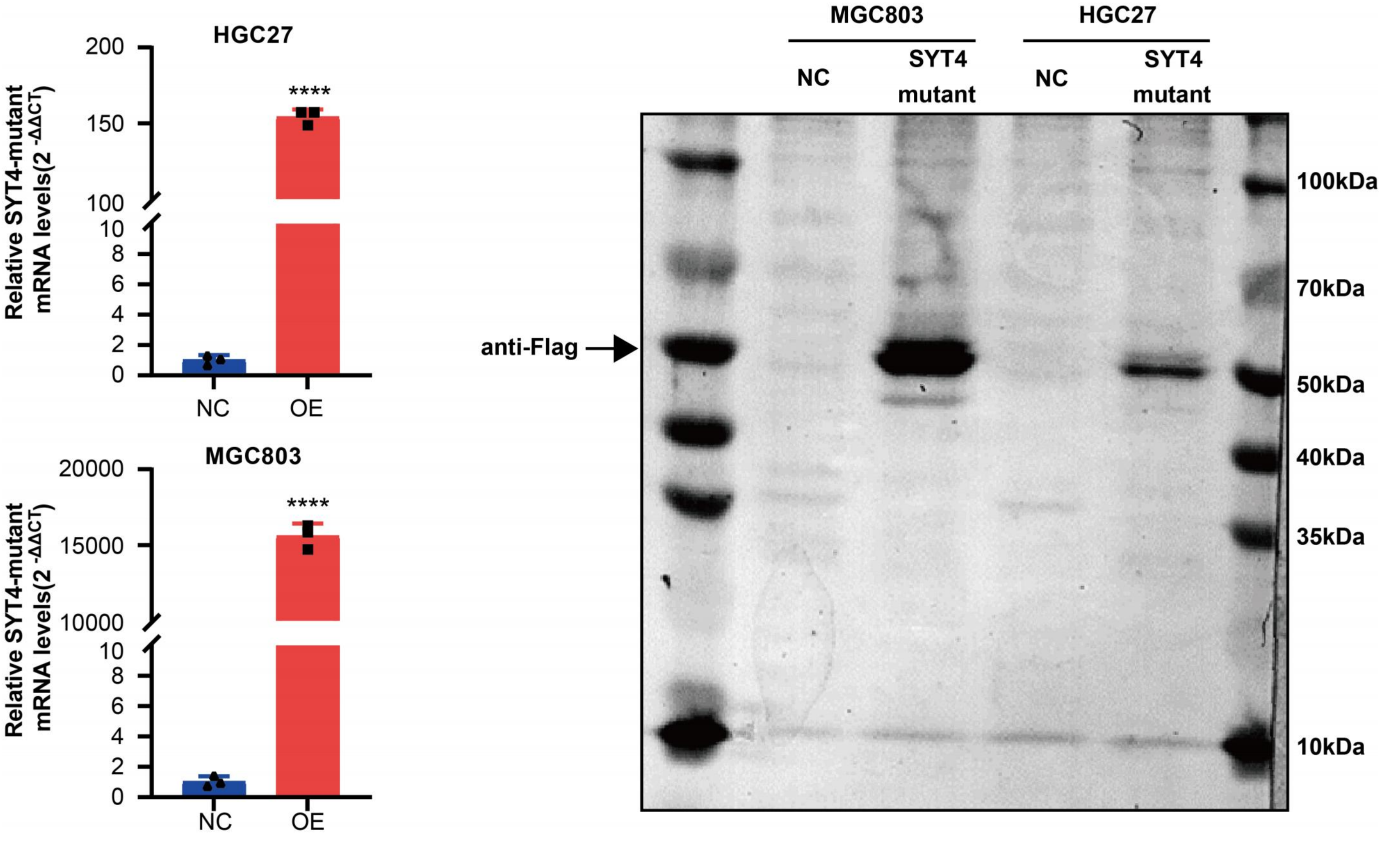

HGC27
Relative SYT4-mutant mRNA levels(2 -ΔΔCT)
200
150
100
10
8
6
4
2
0
****
NC
OE
MGC803
Relative SYT4-mutant mRNA levels(2 -ΔΔCT)
20000
15000
10000
10
8
6
4
2
0
****
NC
OE
MGC803
HGC27
NC
SYT4 mutant
NC
SYT4 mutant
anti-Flag
100kDa
70kDa
50kDa
40kDa
35kDa
10kDa

Supplementary Table S1. The target sequences for shRNAs.

gene		target sequences
shRNA-SYT4	1	GCTCGACATCTGCCTAAATCT
	2	GTGAACAAGCTGAGAAATTAT
shRNA-PSMC6	1	CCAGAGTTATTTCAGCGTGTA
SYT4-OE	transcript	NM_020783

Supplementary Table S2. The PCR primer sequences.

Primers name		Sequence (5’-3’)
SYT4	Forward	TCAGGACGGGGTGAGTTACTG
	Reverse	GTCGAGCTTTTAAGACAACCACA
PSMC6	Forward	GGAGGGCTATCAGAACAGATCC
	Reverse	GGCTCGTGCCAAGAGTGTTT
SYT4-mutant	Forward	TCGGGCAGTTAGTCTTGG
	Reverse	TTGGCAATTTGTCTCCTG
GAPDH	Forward	TGCACCACCAACTGCTTA
	Reverse	GGATGCAGGGATGATGTTC
β-ACTIN	Forward	GACCTGACTGACTACCTCATGAAGAT
	Reverse	GTCACACTTCATGATGGAGTTGAAGG

Supplementary Table S3. Antibodies used for IHC, Western blot, Co-IP and pull-down.

Reagent	Supplier	Catalog number	RRID number
SYT4-antibody	Invitrogen	PA5-87026	AB-2803780
PSMC6-antibody	Abcam	Ab186858	AB-10905872
Ki-67-antibody	Proteintech	27309-1-AP	AB-2756525
AKT-antibody	Abcam	Ab8805	AB-306791
p-AKT (Ser473)-antibody	Abcam	Ab81283	AB-2224551
GSK3β-antibody	Abcam	Ab32391	AB-2115066
GSK3β(phosphor S9)- antibody	Abcam	Ab75814	AB-1310289
β-catenin-antibody	Abcam	Ab32572	AB-725966
Anti-Flag Antibody	Abcam	Ab205606	AB-2916341
Anti-6×His Tag antibody	Proteintech	66005-1-Ig	AB-11232599
Anti-GST-tag antibody	Proteintech	10000-0-AP	AB-11042316
β-ACTIN	Proteintech	66009-1-Ig	AB-2687938
